# Relationships between histogram analysis of ADC values and complex 18F-FDG-PET parameters in head and neck squamous cell carcinoma

**DOI:** 10.1371/journal.pone.0202897

**Published:** 2018-09-06

**Authors:** Hans-Jonas Meyer, Sandra Purz, Osama Sabri, Alexey Surov

**Affiliations:** 1 Department of Diagnostic and Interventional Radiology, University of Leipzig, Leipzig, Germany; 2 Department of Nuclear Medicine, University of Leizig, Leipzig, Germany; Banner Alzheimer's Institute, UNITED STATES

## Abstract

**Purpose:**

Histogram analysis is an emergent imaging technique to further analyze radiological images and to obtain imaging biomarker. In head and neck cancer, MRI and PET are routinely used in clinical practice. The aim of this study was to analyze associations between histogram based ADC parameters and complex FDG-PET derived parameters in head and neck squamous cell carcinoma (HNSCC).

**Methods:**

34 patients (26% female, mean age, 56.7 ± 10.2 years) with primary HNSCC were prospectively included into the study. ADC histogram parameters were calculated by inhouse made matlab software using a whole lesion measurement. For each tumor, maximum and mean standardized uptake values (SUVmax, SUVmean), Total Lesion Glycolysis (TLG) and Metabolic Tumor Volume (MTV) were determined on PET-images. Spearman's correlation coefficient (ρ) was used to analyze associations between investigated parameters. Benjamini-Hochberg correction was used to adjust for multiple testing. Mann-Whitney test was used for group discrimination. P-values < 0.05 were taken to indicate statistical significance.

**Results:**

The correlation analysis in the whole tumor group revealed a statistically significant correlation between entropy and MTV as well as TLG (ρ = 0.67, P<0.0001 and ρ = 0.61, P = 0.0002 respectively).

There were statistically significant differences between T1/2 and T3/4 tumors in the following parameters: entropy (2.07 ± 0.36 vs 2.61 ± 0.43, P = 0.007), SUVmax (10.79 ± 4.13 vs 17.93 ± 5.89, P = 0.007), SUVmean (6.39 ± 2.48 vs 9.81 ± 4.49, P = 0.01), SUVmin (4.09 ± 1.57 vs 6.34 ± 2.59, P = 0.03), MTV (9.50 ± 7.92 vs 20.36 ± 13.30, P = 0.02), TGU (55.97 ± 39.09 vs 212.3 ± 186.3, P = 0.002).

**Conclusion:**

This study showed that entropy derived from ADC maps is strongly associated with MTV and TLG in HNSCC. Entropy, SUVmax, SUVmean, TLG and MTV were statistically significant higher in T3/4 tumors in comparison to T1/2 carcinomas.

## Introduction

Head and neck squamous cell carcinoma (HNSCC) is one of the most frequent malignancies [1]. Different imaging modalities like computed tomography (CT) and magnetic resonance tomography (MRI) are used for correct tumor staging in HNSCC [2]. Nowadays, also functional imaging modalities, such as diffusion-weighted imaging (DWI) can be added into imaging protocol to provide further insight into tumor microstructure [2]. Thus, DWI measures random water movement and can be quantified by the apparent diffusion coefficient (ADC) [3]. Previously, various studies identified an inverse relationship between ADC values and cellularity as well proliferation index indicating that ADC values reflect tumor microstructure [3, 4]. Furthermore, ADC can also predict tumor response to radiotherapy [5] as well tumor behavior, such as disease-free interval in HNSCC [6]. Recently, a novel approach, namely histogram analysis of different images, was proposed [7]. For this technique, every voxel of a region of interest is used to issue a histogram and therefore gain more data regarding tumor [7]. Thereby, a broad spectrum of ADC parameters can be estimated: ADC percentiles, mode ADC, median ADC, kurtosis, skewness, and entropy [7].

Another important functional imaging modality is positron emission tomography with 2-deoxy-2 [18F] fluoro-D-glucose (FDG-PET), which measures glucose metabolism and can be quantified by the standardized uptake value (SUV) [2].

Previously, only few studies investigated possible associations between the functional imaging modalities DWI and FDG-PET with inconclusive results [8–15]. Only one study could identify an inverse correlation between PET and ADC parameters [11], whereas the most did not [9, 12, 14]. However, all of these studies used a conventional ROI-measurement of the ADC values and might, therefore, not be able to identify possible associations. We hypothesize that use of more PET and DWI parameters can show more relationships between glucose metabolism and tissue microstructure. The purpose of this study is to elucidate relationships between ADC histogram parameters and FDG-PET parameters in HNSCC.

## Material and methods

This prospective study was approved by the institutional review board (Ethic comitee of the university of Leipzig, study codes 180–2007, 201-10-12072010, and 341-15-05102015). All methods were performed in accordance with the relevant guidelines and regulations. All patients gave their written informed consent.

### Patients

Overall, 34 patients with primary HNSCC of different localizations were involved in the study (Table 1). There were 9 (26%) women and 25 (74%) men with a mean age of 56.7 ± 10.2 years, range 33–77 years. The identified tumors were localized in the tonsil (n = 8, 23.6%), followed by oropharynx (n = 7, 20.6%), tongue (n = 7, 20.6%), hypopharynx (n = 6, 17.6%), larynx (n = 5, 14.6%), and epipharynx (n = 1, 2.9%). Low and moderately (G1/2) differentiated tumors were diagnosed in 13 cases (38.2%), and high grade (G3) tumor in 21 (61.8%) patients. The diagnosed carcinomas were staged as T1 (n = 1, 2.9%), T2 (n = 7, 20.6%), T3 (n = 10, 29.4%) or T4 tumors (n = 16, 47.1%) with additional nodal (n = 28, 91.2%) metastases. Distant metastases (M) were observed in 4 (11.8%) cases.

**Table 1:** Demographic overview about the patient sample.

**Diagnosis**	**n (%)**
Carcinoma of epipharynx	1 (2.9)
Carcinoma of oropharynx	7 (20.6)
Carcinoma of hypopharynx	6 (17.6)
Carcinoma of larynx	5 (14.7)
Carcinoma of tongue	7 (20.6)
Tonsillar carcinoma	8 (23.6)
**Tumor grade**	**n (%)**
G1/2	13 (38.2)
G3	21 (61.8)
**Tumor stage**	**n (%)**
T1	1 (2.9)
T2	7 (20.6)
T3	10 (29.4)
T4	16 (47.1)
N0	3 (8.8)
N1	6 (17.7)
N2	22 (64.7)
N3	3 (8.8)
M0	30 (88.2)
M1	4 (11.8)

### PET/CT

In all patients an ^18^F-FDG-PET/CT (Siemens Biograph 16, Siemens Medical Solutions, Erlangen, Germany) was performed from the skull to the upper thigh after a fasting period of at least 6 hours. Application of ^18^F-FDG was performed intravenously with a body weight-adapted dose (4MBq/kg, range: 168–427 MBq, mean ± std: 279 ± 60 MBq). PET/MR image acquisition started on average 91 minutes (range 60–270 minutes) after ^18^F-FDG application. In 3/34 patients a PET/MRI scan was performed prior to PET/CT and in 1/34 a technical defect led to a delayed acquisition start, which explains the late PET/CT image acquisition time in these 4 patients. Low-dose CT was used for attenuation correction of the PET-Data.

**Fig 1:**
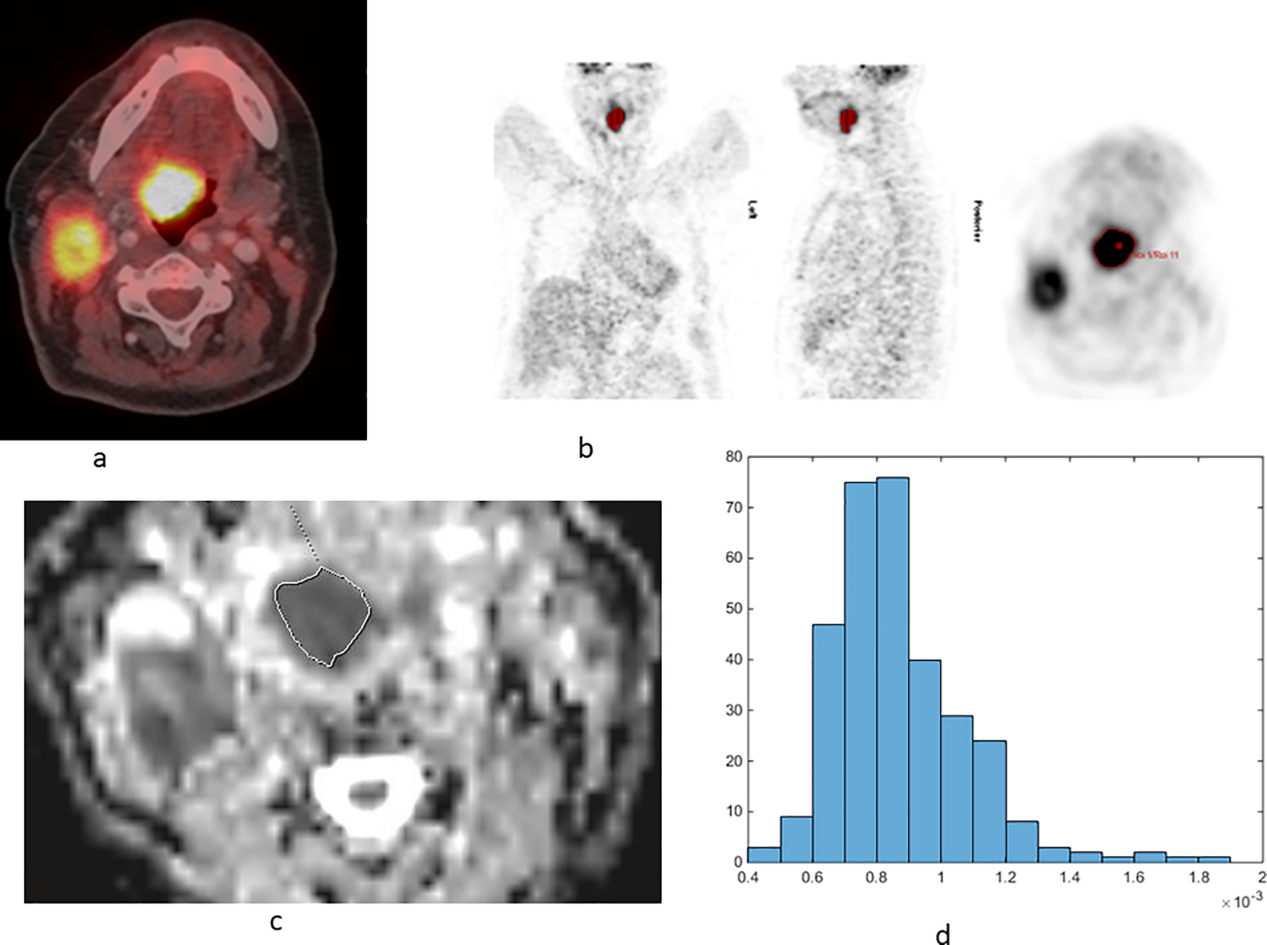
Imaging findings in a 67 year old woman with cT3 cN2b cM0-oropharyngeal carcinoma. a. Fused PET/CT image shows enhanced glucose metabolism in the main tumor as well as in lymph node metastases cervical. b. A polygonal VOI, that include the entire lesion in the axial, sagittal and coronal planes, was placed in the PET dataset (SUV_max_ threshold 40%). The acquired PET parameters are as follows: SUV_max_ = 16.82, SUV_mean_ = 10.01, SUV_min_ = 6.56, MTV = 10.5, and TLG = 105.03. c. ADC map of the lesion. d. ADC histogram. The histogram analysis parameters (× 10^−3^ mm^2^s^-1^) are as follows: ADC_min_ = 0.41, ADC_mean_ = 0.87, ADC_max_ = 1.85, P10 = 0.65, P25 = 0.73, P75 = 0.97, P90 = 1.13, median = 0.83, mode = 0.76, kurtosis = 6.24, skewness = 1.37, and entropy = 2.93.

The acquired PET/CT datasets were evaluated by a board certified nuclear medicine and a board certified radiologist with substantial PET/CT experience in oncological image interpretation. PET/CT image analysis was performed on the dedicated workstation of Hermes Medical Solutions, Sweden. For each tumor, maximum and mean SUV (SUV_max_ and SUV_mean_) were calculated. Furthermore, Total Lesion Glycolysis (TLG) and Metabolic Tumor Volume (MTV) were determined on PET-images. Prior to this, tumor margins of the HNSCC were identified on diagnostic CT images and fused PET/CT images and a polygonal volume of interest (VOI), that include the entire lesion in the axial, sagittal and coronal planes, was placed in the PET dataset (SUV_max_ threshold 40%), see Fig 1A and 1B. MTV was defined as total tumor volume with an SUV ≥ 2.5 and was calculated automatically. TLG was also calculated automatically by multiplying the MTV of the primary tumor by its SUV_mean_.

### MR imaging

In all patients, neck MRI was performed using a combined head and neck coil. The imaging protocol included an axial T1 weighted (T1w) turbo spin echo (TSE) sequence prior and after intravenous application of contrast medium (Gadovist®, Bayer Healthcare, Leverkusen, Germany), with a dose of 0.1 mmol per kg of body weight, an axial T2 weighted (T2w) fat-supressed short tau inversion recovery (STIR) sequence. DWI was obtained using an EPI (echo planar imaging) sequence (TR/TE: 8620/73 ms, slice thickness: 4 mm, and voxel size: 3.2 x 2.6 x 4.0 mm) with b-values of 0 and 800 s/mm^2^.

### ADC histogram analysis

For each tumor, automatically generated ADC maps were saved in DICOM format and processed offline with custom-made Matlab-based application (The Mathworks, Natick, MA) on a standard windows operated system. Polygonal regions of interest (ROI) were manually drawn on the transferred ADC maps along the contours of the primary tumor on each slice (whole lesion measure) (Fig 1C). All measures were performed by one radiologist (A.S., 15 years radiological experience). The following parameters were calculated (Fig 1D): mean ADC (ADC_mean_), maximum ADC (ADC_max_), minimum ADC (ADC_min_), median ADC (ADC_median_), mode ADC (ADC_mode_). Furthermore, ADC percentiles: 10^th^ (P10 ADC), 25^th^ (P25 ADC), 75^th^ (P75 ADC), and 90^th^ (P90 ADC), as well histogram-based characteristics of the ROIs—kurtosis, skewness, and entropy–were estimated [16].

### Statistical analysis

Statistical analysis and graphics creation was performed using Graph Pad Prism package (GraphPad Software, La Jolla, CA, USA). Collected data were evaluated by means of descriptive statistics. Spearman's correlation coefficient (ρ) was used to analyze associations between investigated parameters. Benjamini-Hochberg correction was used to adjust for multiple testing. Mann-Whitney test was used for group discrimination. P-values < 0.05 were taken to indicate statistical significance.

## Results

**Fig 2:**
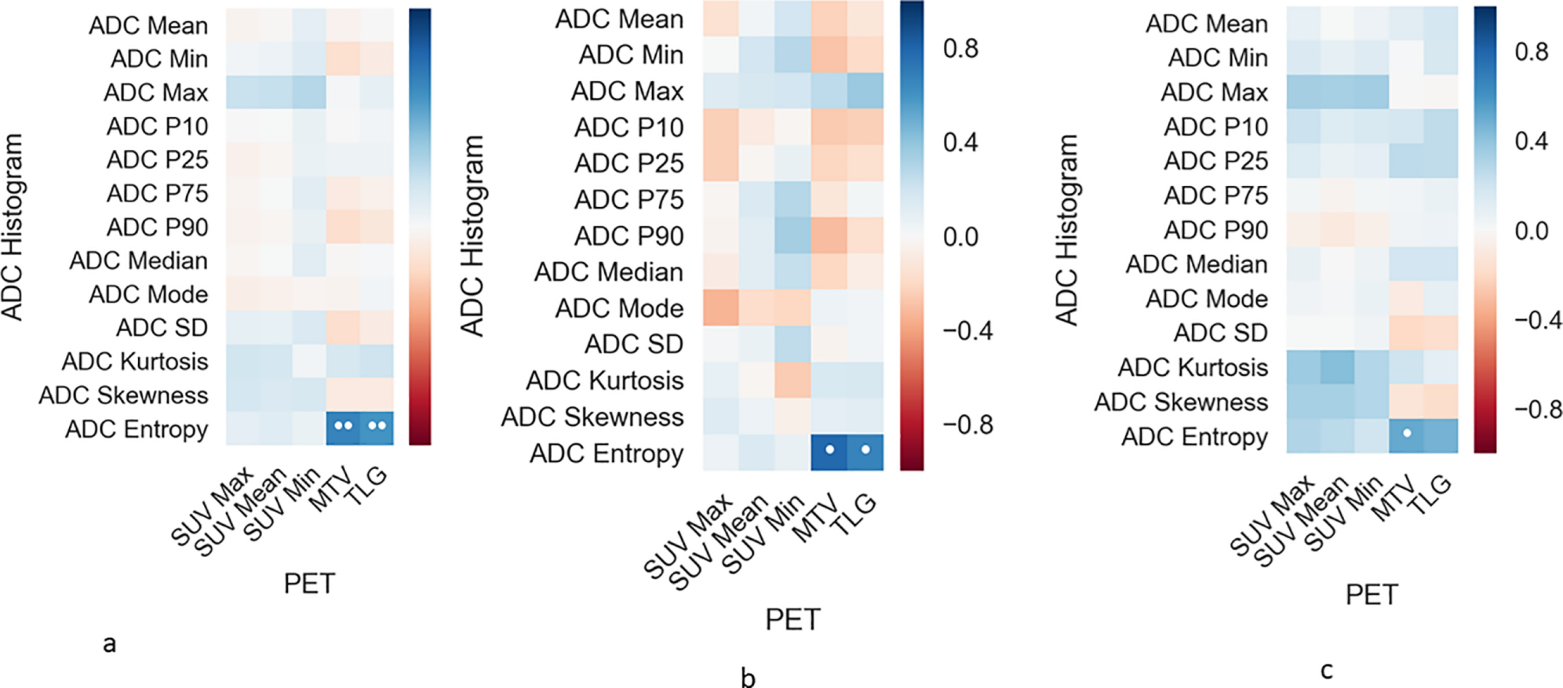
Correlation heat map in the overall sample (a). b. summarizes the correlations of well differentiated, c of poor differentiated HNSCC.

The estimated PET and ADC parameters are summarized in Table 2. Fig 2A–2C display the correlation coefficients between PET and ADC parameters as heat maps.

**Table 2:** Overview about the investigated imaging parameters.

Parameter	M ± SD	Range
**ADC mean**	1.13 ± 0.20	0.78–1.68
**ADC min**	0.69 ± 0.22	0.17–1.24
**ADC max**	1.76 ± 0.31	1.35–2.39
**P10**	0.89 ± 0.19	0.54–1.42
**P25**	0.98 ± 0.19	0.64–1.49
**P75**	1.25 ± 0.22	0.87–1.82
**P90**	1.40 ± 0.25	0.94–2.03
**Median**	1.10 ± 0.20	0.76–1.64
**Mode**	0.97 ± 0.28	0.78–1.55
**Kurtosis**	3.62 ± 1.39	1.91–7.93
**Skewness**	0.50 ± 0.44	-0.20–1.49
**Entropy**	2.49 ± 0.46	1.70–3.75
**SUVmax**	16.37 ± 6.25	5.90–35.56
**SUVmean**	9.59 ± 3.94	3.63–21.74
**SUVmin**	5.85 ± 2.45	2.22–12.79
**MTV**	17.98 ± 13.03	2.41–47.29
**TLG**	178.1 ± 177.4	16.20–866.4

**Fig 3:**
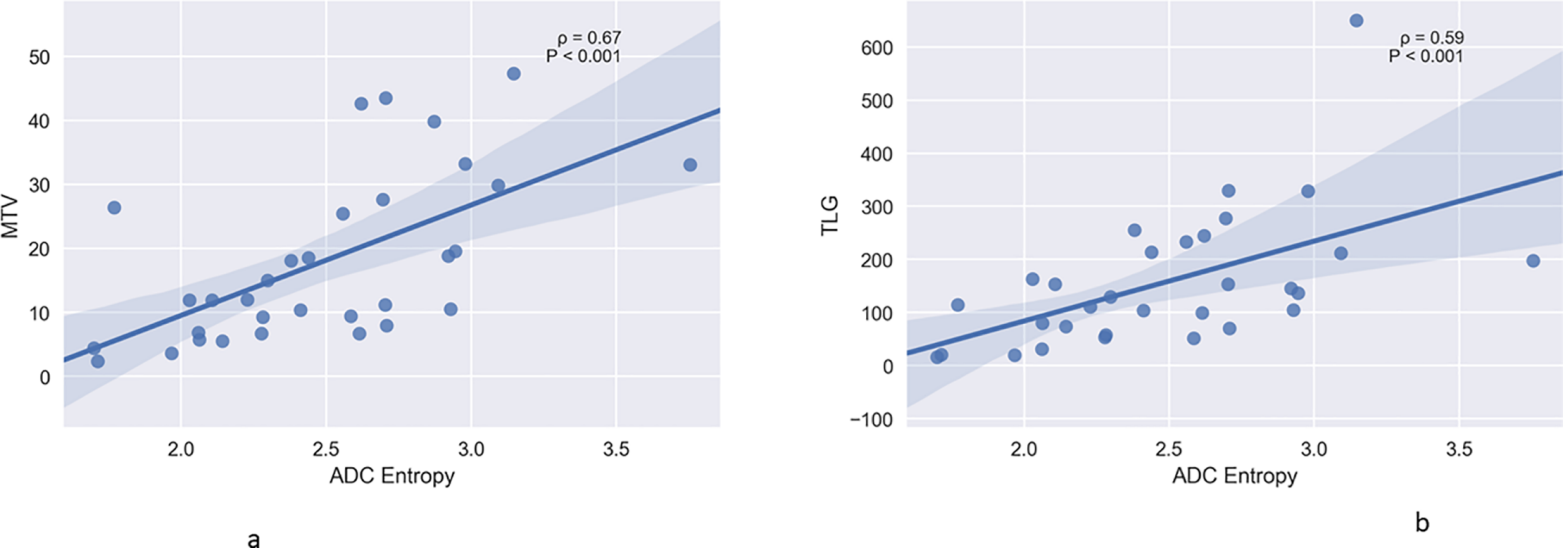
a. Associations between MTV and ADC entropy in the overall sample (ρ = 0.67, P<0.0001). b. Correlation between TLG and ADC entropy in the overall sample (ρ = 0.61, P = 0.0002).

In the overall sample, statistically significant correlations between ADC entropy and MTV (ρ = 0.67, P<0.0001) and TLG (ρ = 0.61, P = 0.0002) were identified (Fig 3A and 3B).

In well and moderately differentiated tumors alone, the correlations between ADC entropy and MTV and TLG were stronger. For poor differentiated tumors the correlations were weaker. However no correlation reached statistically significance after correction for multiple testing.

There were no statistically significant differences between the analyzed parameters in G1/2 and G3 tumors (Table 3).

**Table 3:** Comparison of the analyzed parameters between well/moderately and poor differentiated tumors.

Parameter	G1 M ± SD	G3 M ± SD	P-value (Mann- Whitney test)
ADC mean	1.16 ± 0.14	1.11 ± 0.24	0.21
ADC min	0.74 ± 0.15	0.65 ± 0.26	0.30
ADC max	1.75 ± 0.25	1.78 ± 0.35	0.79
P10	0.93 ± 0.13	0.85 ± 0.22	0.08
P25	1.03 ± 0.13	0.95 ± 0.22	0.07
P75	1.27 ± 0.15	1.24 ± 0.26	0.38
P90	1.43 ± 0.19	1.39 ± 0.28	0.49
Median	1.14 ± 0.14	1.08 ± 0.24	0.14
Mode	1.01 ± 0.19	0.95 ± 0.34	0.44
Kurtosis	3.39 ± 0.96	3.77 ± 1.63	0.79
Skewness	0.49 ± 0.31	0.50 ± 0.52	0.88
Entropy	2.59 ± 0.45	2.42 ± 0.48	0.13
SUVmax	16.01 ± 4.89	16.62 ± 7.16	0.76
SUVmean	9.28 ± 3.11	9.81 ± 4.49	0.85
SUVmax/mean	1.75 ± 0.25	1.72 ± 0.21	0.70
SUVmin	5.62 ± 1.97	6.00 ± 2.78	0.67
MTV	19.68 ± 14.60	16.82 ± 12.12	0.55
TLG	188.4 ± 171.1	171.0 ± 186	0.70

**Fig 4:**
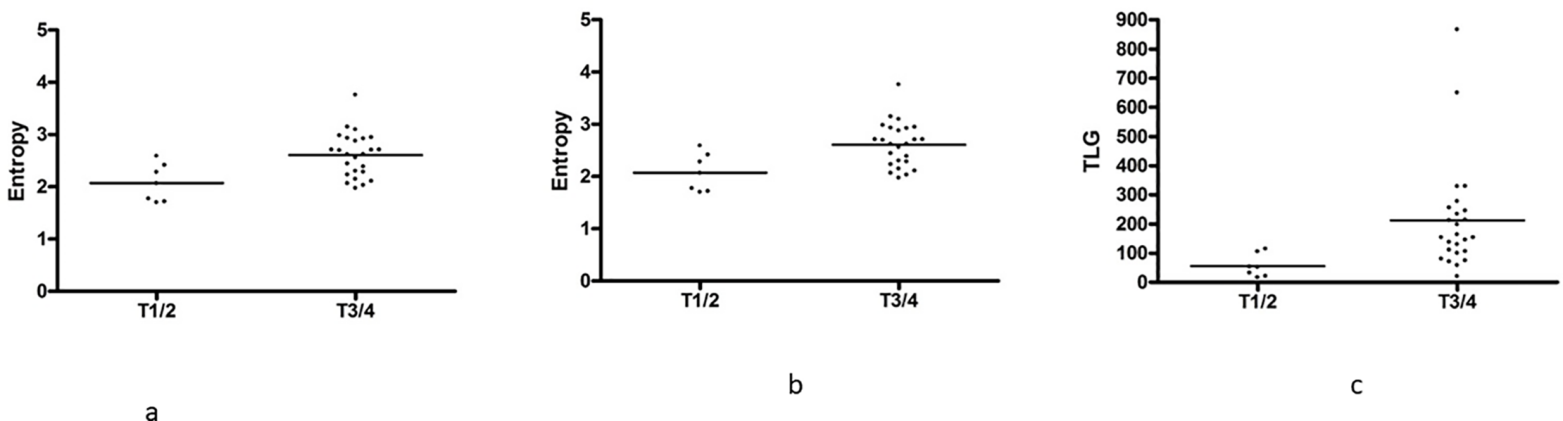
a. Comparison of ADC entropy between different tumors. ADC entropy is statistically significant higher in T3/4 than T1/2 tumors (2.61 ± 0.43 vs 2.07 ± 0.36, p = 0.007). b. Comparison of SUVmax between different tumors. SUVmax is statistically significant higher in T3/4 than in T1/2 tumors (17.93 ± 5.89 vs 10.79 ± 4.13, p = 0.007). c. Comparison of TLG between different tumors. TLG is higher in T3/4 than in T1/2 tumors (212.3 ± 186.3 vs 55.97 ± 39.09, p = 0.002).

There were statistically significant differences between T1/2 and T3/4 tumors in the following parameters: entropy (2.07 ± 0.36 vs 2.61 ± 0.43, P = 0.007), SUVmax (10.79 ± 4.13 vs 17.93 ± 5.89, P = 0.007), SUVmean (6.39 ± 2.48 vs 9.81 ± 4.49, P = 0.01), SUVmin (4.09 ± 1.57 vs 6.34 ± 2.59, P = 0.03), MTV (9.50 ± 7.92 vs 20.36 ± 13.30, P = 0.02), TGU (55.97 ± 39.09 vs 212.3 ± 186.3, P = 0.002) (Table 4, Fig 4A–4C).

**Table 4:** Comparison of the analyzed parameters between T1/2 and T3/4 tumors.

Parameter	T1/2 M ± SD	T3/4 M ± SD	P-value (Mann- Whitney test)
**mean**	1.05 ± 0.15	1.11 ± 0.21	0.39
**min**	0.60 ± 0.21	0.72 ± 0.23	0.24
**Max**	1.69 ± 0.33	1.79 ± 0.30	0.41
**P10**	0.78 ± 0.15	0.92 ± 0.19	0.13
**P25**	0.88 ± 0.16	1.01 ± 0.20	0.19
**P75**	1.20 ± 0.18	1.27 ± 0.23	0.62
**P90**	1.40 ± 0.23	1.41 ± 0.26	0.58
**Median**	1.02 ± 0.14	1.13 ± 0.21	0.26
**Mode**	0.79 ± 0.34	1.03 ± 0.25	0.12
**Kurtosis**	3.26 ± 1.00	3.72 ± 1.48	0.49
**Skewness**	0.45 ± 0.42	0.51 ± 0.45	0.98
**Entropy**	**2.07 ± 0.36**	**2.61 ± 0.43**	**0.007**
**SUVmax**	**10.79 ± 4.13**	**17.93 ± 5.89**	**0.007**
**SUVmean**	**6.39 ± 2.48**	**9.81 ± 4.49**	**0.01**
**SUVmax/mean**	1.69 ± 0.05	1.74 ± 0.25	0.62
**SUVmin**	**4.09 ± 1.57**	**6.34 ± 2.59**	**0.03**
**MTV**	**9.50 ± 7.92**	**20.36 ± 13.30**	**0.02**
**TLG**	**55.97 ± 39.09**	**212.3 ± 186.3**	**0.002**

Significant differences are highlighted in bold

## Discussion

This present study identified several statistically significant associations between PET and ADC histogram analysis parameters in HNSCC. In particular, strong correlations between ADC entropy and MTV and TLG were observed.

In a recent meta-analysis a weak inverse correlation coefficient (r = -0.30) was estimated between ADC values and SUV in neoplastic lesions [17]. Moreover, in HNSCC, the calculated correlation coefficient was weaker, namely r = -0.27 [17]. It has been suggested that both modalities might reflect different tumor aspects. DWI is widely acknowledged to reflect cellularity and proliferation activity [3, 18], whereas FDG-PET is mainly influenced by glucose transporters [19]. Especially GLUT-1 and Glut-3 are important in tumors [19]. Presumably, tumors with more dense packed cells might also express more GLUT transporters and, therefore, both imaging modalities might be linked to each other. However, previously, most authors investigated associations between different parameters of PET and DWI in HNSCC did not find significant correlations between ADC and parameters of glucose metabolism [9, 10, 12, 13]. The cause of this phenomenon is unclear. One of possible reasons might be the fact that the reported studies involved different carcinoma groups, namely primary and recurrent tumors and/or several tumor grades. This assumption can be confirmed by some recent publications. For instance, Leifels et al. suggested that associations between metabolism, water diffusion and perfusion in HNSCC depend on tumor grade [15]. Furthermore, Covello et al. identified no significant correlations between PET parameters and ADC values in primary carcinomas, but found a strong correlation (r = -0.72, p = 0.01) between ADCmean and SUV in patients with recurrent tumors [9]. Thus, it is to note to strictly divide patient samples with primary and recurrent tumors in these correlation studies because they may show different tumor biology behavior. In addition, most previous reports investigated only associations between routinely used parameters, i.e. mean ADC values and SUVmax [8–15]. Presumably, these parameters may be not sensible to show all relationships between metabolic activity and tissue architecture. In fact, Han et al. identified an inverse correlation between ADCmin and TLG (*r* = −0.347, *P* = 0.04), suggesting associations between glucose metabolism and cellularity [8].

The present study involved only primary tumors. Furthermore, complex ADC and PET parameters were acquired. Additionally, associations between the imaging findings were analyzed separately in different tumor groups. Overall, our study identified the following. Firstly, it showed that conventional ADC values and PET parameters had no statistically significant correlations, as it was previously shown. Secondly, as a new aspect regarding functional imaging, the present study identified strong positive correlations between ADC entropy and PET parameters MTV and TLG. On the one hand, this finding confirms the assumption that associations between tumor metabolism and tissue microstructure in HNSCC are linked to each other. On the other hand, it suggests that conventional ADC and PET parameters used in clinical practice do not reflect these relationships. Thirdly, we found that correlations between the analyzed PET and ADC parameters were stronger in low/moderate grade tumors than in G3 lesions.

Interestingly, only entropy of ADC values correlated significantly with PET parameters. Recently, entropy as a novel histogram based parameter has been increasingly acknowledged to be a very promising biomarker [7, 20–22]. In short, entropy represents the heterogeneity of the histogram and is therefore believed to reflect also heterogeneity of tumor microstructure [7]. For example, in cervical cancer entropy was significantly higher in T3/4 tumors than in lower tumor stages [23]. Moreover, entropy was significantly associated with p53 expression in cervical cancer [22]. Additionally, entropy can discriminate pancreatic ductal carcinoma and neoendocrine tumors [24].

Regarding MTV and TLG, they are somewhat established and previously extensively researched parameters. In a recent meta analysis it could be identified that MTV and TLG had a higher hazard ratio than SUVmax for predicting event free survival in nasopharygeal carcinomas [25]. Moreover, MTV was stronger associated with overall survival than SUVmax [25]. Furthermore, MTV was an indepent predictor for relapse-free survival in multi-variate analysis in HNSCC [26].

As a limitation of the present study is to mention that the patient sample is relatively small and therefore no adjustment for possible confounding factors could be performed.

The present study could not identify differences in imaging parameters between poorly and well/moderately differentiated carcinomas, which is in good agreement with the literature [10]. However, the present study showed that some imaging parameters were different in different tumor stages. Especially ADC entropy can discriminate T3/4 and T1/2 lesions. Presumably, in larger tumors such as T3/4 there are also more different microenvironments, which can be reflected by ADC entropy. Furthermore, also SUVmax was higher in advanced cancers. In concordance with our study, Nakajo et al showed that T3/4 tumors have higher SUVmax values than T1/2 tumors [11].

## Conclusions

This study showed that entropy derived from ADC maps is strongly associated with MTV and TLG in HNSCC. This correlation was even stronger in G1/2 tumors. Additionally, entropy, SUVmax, SUVmean, TLG and MTV were statistically significant higher in T3/4 tumors in comparison to T1/2 carcinomas.
